# The Affective Domain, Safety Attitude, and COVID-19 Prevention of Employees in the Petrochemical Industry

**DOI:** 10.3390/bs13050380

**Published:** 2023-05-04

**Authors:** Gwo-Long Lai, I-Jyh Wen, Wei-Liang Chien

**Affiliations:** 1 Department of Civil and Construction Engineering, National Yunlin University of Science and Technology, Douliu 640301, Taiwan; 2 Graduate School of Engineering Science and Technology, National Yunlin University of Science and Technology, Douliu 640301, Taiwan

**Keywords:** affective domain, safety attitude, COVID-19 prevention, petrochemical industry workplace

## Abstract

The petrochemical industry is relatively strict regarding safety rules in the workplace. The workplace involves high-risk categories that are intolerant of human error. Especially in the current situation with COVID-19, concerns regarding prevention and safety in the workplace have increased. In light of this pandemic, the company must know whether all employees recognize the implementation of COVID-19 prevention. In addition, employee awareness of safety grounded in the affective domain of human thought is lacking. This study investigates the safety attitudes and COVID-19 prevention in the workplace based on the affective domain of employees. A survey questionnaire based on the Likert scale was utilized to collect data from 618 employees in the petrochemical industry. Descriptive analysis and analysis of variance were used to examine the data. The results reveal that employees in the petrochemical industry have a positive degree of responses to COVID-19 prevention, safety attitudes, and the affective domain, regardless of employment characteristics such as gender, age, position, and work experience. This study concludes that a positive affective domain of employees is followed by a positive safety attitude; thus, effective COVID-19 prevention was established in the workplace based on the perspectives and attitudes of the employees.

## 1. Introduction

The petrochemical industry is considered to consist of complex and high-risk tasks and processes, such that human error can have catastrophic consequences, including death, severe economic loss, and widespread environmental pollution [1]. Therefore, the slightest human error is not tolerated [2]. In addition, many chemicals used during petrochemical manufacturing are toxic, flammable, and pyrophoric [3]. These issues endanger the safety of workers and the work area. However, many accidents have been caused by repeating the same human error or improper behaviors.

Safety and environmental protections are higher priorities for petrochemical plants than those of the general construction industry [4]. Therefore, the construction personnel of the petrochemical plant are operating under relatively high pressure. The safety of each element of the petrochemical industry must be guaranteed, which is dependent on employee behaviors in the workplace.

Safety at work is a combination of competency and attitude. In general, accidents at work occur either due to a lack of supervision, a lack of knowledge or training, or a lack of means to carry out the task safely [5]. In addition to these factors, the short-term and transitory nature of the petrochemical industry, the lack of a controlled workplace, and the complexity and diversity of organizational procedures will influence safety performance within the industry [6].

Given the current situation, many countries have fought against the massive spread of the coronavirus (COVID-19) by establishing social distancing as a public policy and implementing screening tests in many social activities [7]. Public policy has been published with the priority focused on people’s health. COVID-19 directly affects the physical health and emotional and social behavior of all citizens [8].

The petrochemical industry is considered to have high health and safety standards, especially in light of the implementation of COVID-19 prevention in the workplace [9]. Although COVID-19 transmission can drastically impact the health and safety of employees in the workplace, the petrochemical industry sector still needs to execute construction projects during the COVID-19 pandemic, including maintaining equipment and employing process safety guidelines to prevent a catastrophic petrochemical event [10]. 

Therefore, this study aims to determine the level of COVID-19 prevention and safety attitude based on the workers’ affective domain. To achieve this goal, this research proposes the following research questions:

1. What are the workers’ perspectives on COVID-19 prevention, safety attitude, and affective domain in the petrochemical industry?
2. What are the workers’ views on COVID-19 prevention, safety attitude, and the affective domain according to their gender, age, position, and work experience in the petrochemical industry?
3. What are the connections between COVID-19 prevention, safety attitude, and affective domain based on the data obtained?

## 2. Fundamental Theories

### 2.1. Affective Domain

The affective domain involves our attitudes, feelings, and emotions, or, in other words, how we deal with things emotionally [11]. The emotions themselves are included in our attitudes, motivations, enthusiasm, appreciation, values, and feelings. The affective domain is arranged in a hierarchy whereby people have complex feelings that result from simpler feelings, according to the definition given by Bloom [10]. One of the guiding principles for the evolution of feelings through the hierarchy is internalization. Internalization is the process whereby people’s feelings toward something go from a natural state of awareness to a point of effect [12]. The affect feeling is internalized and consistently guides, controls, and gives feedback on our behavior. Based on that, the affective domain is the set of actions that are produced from and influenced by emotions. Consequently, the affective domain involves attitudes, values, appreciations, emotions, and behaviors [13]. An employee also demonstrates behaviors in the workplace, indicated by attitudes toward responsibility, interest, concern, and attention in daily work activities [14]. Most psychologists and researchers describe five levels within the affective domain that outline the path from passively observing a stimulus to affecting our actions.

1. Receiving: Being aware of something in the workplace environment. Awareness of employees includes the need and willingness to hear selected attention, e.g., listening respectfully to others, listening, and remembering rules in the workplace;
2. Responding: Performing some new behaviors as a result of work experience. Active participation at work includes responding to various tasks. Working outcomes may result in satisfaction or increased motivation, willingness to respond, or enhanced compliance;
3. Valuing: Showing some definite involvement or commitment in the working environment, and having the ability to judge the worth or value of a task, including specific tasks, behaviors, information, or phenomena, and to express it clearly from a simple state of commitment to a complex state of commitment;
4. Organization: Integrating a new value into one’s general set of values or giving it some ranking among one’s general priorities in a task. A more specific definition is resolving conflicts, comparing and classifying values, and creating a unique value system (with a focus on relevance, comparison, and integrated values);
5. Characterization: Acting consistently with the new value. Acting consistently is defined as the establishment of a value system that controls human behavior, which is a predictable, consistent, universal, and prominent feature of humans.

### 2.2. Safety Attitude

The petrochemical industry is distinct from other manufacturing industries, including several unique characteristics such as a temporary organizational structure, complicated construction processes, changing work locations, high-stress work conditions, and complex interactions with various environments [15]. The focus on improving interactions between humans and the environment will decrease the probability of the occurrence of hazards and accidents in the workplace. For instance, the prevention of accidents can be achieved by the constant daily monitoring of operations and activities to ensure the smooth and safe functioning of the whole chemical process [16]. One assessment that can be utilized is safety attitude monitoring. Safety attitude measures the firms’ preparedness in controlling and preventing incidences and setting safety targets and goals for reducing fatalities caused by hazards [17].

Much attention is given to the issue of human error, including the fundamental causes of human error and accidents in safety-critical systems, such as unsafe conditions and unsafe behavior [18]. While unsafe conditions can be attributed to technology-related factors, such as inadequate technology design and poorly maintained equipment, unsafe behavior depends on personal safety attitudes [19]. A safety attitude is a necessary concept for maintaining and optimizing safe workplaces. This can be attained through organizational safety culture and the safety behaviors of workers, or the ability to prevent and eliminate accidents or occupational injuries [20].

### 2.3. COVID-19 Prevention

The transmission of COVID-19 occurs via coughing and contaminated hands and surfaces [21]. However, the close transmission or direct contact with COVID-19 can be reduced by shifting human habits [22]. Concerning safety and health measures for controlling and preventing the spread of COVID-19, the most frequent measures include the implementation of remote work and staggered breaks and lunches. In addition, many firms have separated project offices to reduce the number of onsite workers. Hence, considering the global emergency of COVID-19 infections, preventive care is necessary to restrict its transmission. Infections associated with coronavirus may spread by contact, the airborne transmission of droplets, and inappropriate habits [23]. These transmissions will be minimized by taking appropriate preventive measures and improving good habits in daily activity or working. It is important to take preventive action to reduce infection and mortality rates [24]. Preventive actions taken throughout the pandemic also include self-imposed measures, such as vaccinations, social distancing, wearing masks in public, and good self-hygiene becoming a basic habit.

The pandemic was controlled as more people carried hand sanitizer and wore masks. Social distancing was recommended, especially where there was community transmission, especially in work areas. Many companies implemented physical distancing to prevent the further spread of the virus.

## 3. Methodology

### 3.1. Research Framework

**Figure 1:**
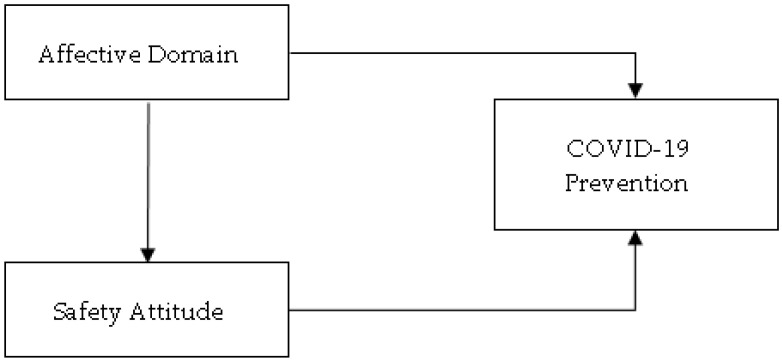
Connection each variable.

Based on the establishment of theories that were mentioned in the literature review, this study speculates that the affective domain impacts human ability, attitude, and behavior in the workplace. Numerous studies have focused on the affective domain in education; however, there has been less attention regarding the affective domain in the workplace. Therefore, the current study built a research framework based on the workplace, as shown in Figure 1. This study considers three important factors that are currently prominent in the workplace, especially those related to safety, including the implementation of COVID-19 prevention, safety attitudes, and the affective domain of employees in the petrochemical industry.

### 3.2. Population Sample

The population sample of this research consisted of employees in one of the petrochemical industries in Taiwan. Sample selection was carried out randomly by emailing electronic questionnaires to all employees. The questionnaire and responses were sent because the researchers cooperated with top management companies; thus, the filling out of this questionnaire had a top-down flow. The respondents included managers, engineers, and construction personnel from various departments. The 770 questionnaires were collected, and 152 incomplete questionnaires were deducted, leaving 618 complete questionnaires. Thus, the questionnaire response rate was 80%.

### 3.3. Data Collection

The online questionnaire was sent by e-mail, requesting that respondents (petrochemical employees) fill out an online form using the Likert scale. This research questionnaire was adopted from published articles with the following details: (1) COVID-19 prevention (according to Card in 2022) [25], with a reliability score of 0.80; (2) safety attitude (according to Ahmed Naji in 2022) [26], with a reliability score of 0.907; and (3) affective domain (according to Camelia in 2018) [27], with a reliability score of 0.908. These questions were answered according to the degree of implementation of the subject in daily work activity: (1) the first part included five questions to measure the degree of implementation of COVID-19 prevention; (2) the second part used nine questions to measure the safety attitude of the employees; and (3) the last part had 20 questions to measure the affective domain of the employees.

### 3.4. Data Analysis

Statistical Product and Service Solutions (SPSS) software was used to analyze statistical nonparametric data, which also included descriptive statistical analysis and a comparison of different tests using analysis of variance. Nonparametric analysis was used because the data results were taken from the questionnaire in 5 Likert scales, so the normality test could not be carried out. In addition, the measurement instruments were adopted from published articles, so validity and reliability were provided in the original articles and rewritten in the data collection. Meanwhile, descriptive statistics were used to obtain frequency responses (grouped into negative, neutral, and positive categories) of each question in the instrument. Further, the Mann–Whitney differential test was used specifically to determine whether there were differences between the two gender groups. At the same time, the Kruskal–Wallis test was used to determine whether there were differences in more than two groups in each respondent’s characteristics (age, position, and work experience).

## 4. Result

### 4.1. Sociodemographic Characteristics of the Participants

Table 1 describes descriptive information of the respondents grouped by gender, age group, job position, and work experience. The data were collected from employees in the petrochemical industry in related fields. Table 1 reveals that, in the petrochemical industry, with high-risk job tasks, males make up the majority of employees.

### 4.2. Employee Responses

Table 2 reveals that the respondents’ answers relating to COVID-19 prevention were positive. COVID-19 affects the economy and the daily life of employees, and the effective prevention and control of the epidemic make up the foundation of rebuilding economic and social development. Respondents realized that controlling the epidemic has the potential to improve economic development and provide favorable conditions for normal life.

To improve the efficiency of the petrochemical industry and reduce downtime due to COVID-19, it is necessary to control the frequency of interaction between employees. The content regarding epidemic prevention methods was effectively recognized by the respondents, particularly questions 3, 4, and 5.

Table 3 reveals that the majority (2/3) of respondents answered in the positive category and the rest answered (1/3) in the neutral category. Positive safety attitudes in the workplace are essential to avoid an accident in the workplace and to ensure that company processes have higher efficiency and save money on the cost of an accident, thus raising employee morale, business profits, and goodwill. A negative attitude toward safety refers to unsafe behaviors. These behaviors lead to incidents occurring on the job, resulting in injury or property damage.

Table 4 shows the answers to questions related to the affective domain of employees, revealing that the majority of respondents answered in the positive category and the “managerial skills and big picture thinking” questions were answered in the neutral category. Since each employee’s management thinking and methods have their own logic, there was no consistent answer in this regard. Most of the respondents are employees, and they were found to seldom consider management work because the operation of equipment is non-stop operation, so employees spend most of their time maintaining petrochemical equipment and operations.

### 4.3. Analysis of Variance

Table 5 and Table 6 show the results of the analysis of variance of each variable based on the employee characteristics. The results indicate that there were no significant differences. This is indicated by the significance value or *p*-value of the analysis results, which was greater than the significance threshold (*p* > 0.05). This means that the implementation of the three variables was successful, regardless of the characteristics.

## 5. Discussion

Based on the results of this study, Table 2, Table 3 and Table 4 reveal that most workers’ responses to COVID-19 prevention, safety attitudes, and affective domains were in a positive category. This explains that petrochemical workers’ awareness of COVID-19 prevention is excellent. The good results also followed this in measuring safety attitudes in petrochemical work. Therefore, the point of view on the two linked theories reveals that workers with good safety in their daily work also implement COVID-19 prevention. Both of these factors are intrinsic to the behavior of company workers. Therefore, this research involves a person’s affective domain to strengthen the relationship between the two factors in worker behavior. The result states that the affective domain of workers is also in line with the outcome of two behavioral factors in positive responses.

Table 5 and Table 6 reveal no differences between categories (groups) in each characteristic of the variable being measured. This explains that awareness of COVID-19 prevention and safety in the petrochemical industry is evenly distributed among industrial petrochemical workers. Gender, position, and work experiences have both good awareness and discipline. This is also consistent when viewed from the perspective of the affective domain, and there is no difference between the categories for each characteristic. In other words, there is a link between the awareness of prevention and the discipline of safety with the affective domain of workers.

If discussed generally, the COVID-19 outbreak has been the largest global health crisis in recent years. It has had a huge impact on workforces and workplaces around the world. In addition, COVID-19 has triggered dramatic changes in the workplace and raised the level of concern among employees about their mental and physical health. The petrochemical industry also has been significantly impacted by the COVID-19 pandemic and faces the challenge of improving the safety and well-being of employees [28].

Employee attitudes toward safety indicate that they generally behave safely in the workplace and accept or adhere to formal workplace rules [29]. In addition, to improve safety, they will take the initiative to implement informal practices where necessary. Past behaviors also affect employee attitudes and safety at work [30]. If someone has the willingness to perform a petrochemical construction task safely, then they will positively improve their knowledge regarding how to properly perform a task and are likely to repeat good behavior. Conversely, when an employee takes shortcuts and performs a task unsafely with little or no consequence, they are likely to continue working with unsafe behavior [31].

Human well-being is generally defined as a multi-faceted construct, including both affective and cognitive aspects. In addition, both aspects are influenced by a person’s psychological, physical, and social resources and life experiences. The COVID-19 pandemic has forced new challenges upon employees, such as uncertainty about job sustainability, which is likely impacting their well-being [32]. Nevertheless, it would be a generalization to assume that employees were negatively influenced and mentally affected by COVID-19. Most likely, there is substantial variance in level of adjustment, where some groups of employees are more vulnerable than others. Therefore, it is necessary to identify psychological factors that can have a crucial role in the mental outcomes of an employee during a crisis.

According to the literature, males and females act the same with regards to COVID-19 prevention in the work area [9]. This is also true when comparing young with old employees, subordinates with supervisors, and new workers with experienced workers. After all, preventive behavior in the workplace is mostly a system established by the company. To implement work safety, all employees will generally need to abide by the rules of the system [33].

Safety attitude is a higher-order concept that is necessary for safety work improvement. The dynamic hazards in petrochemical plants make safety awareness one of the significant factors for the plants’ safety culture [34]. Moreover, regular safety training and specific observation among management can help employees improve safety knowledge, which in turn improves attitudes toward participation in plant safety programs [35]. Continuous improvement has a strong impact on employee safety performance.

Considering the affective domain, males and females usually exhibit similar behaviors in the workplace [36]. This is also the same for young and old employees, subordinates and supervisors, and new and experienced workers. The affective component reflects a human value and how they may feel about a certain object or situation, such as an emotional reaction. These emotions and feelings are transformed by past experiences that may resurface when similar situations or conditions are relived.

Based on the results, we conclude that workers in petrochemical industries exhibit a positive affective domain and safety attitude, and care about COVID-19 prevention [37]. Employees with a positive affective domain will promote safe behaviors in the workplace, and they are more prepared to face the pandemic situation at work or in their daily life.

Employees who have positive emotional domains tend to gain a great deal of satisfaction from daily life and work activities. Positive employees rarely give up [38]. They generally perform well in the workplace because they are encouraged to embrace new ideas, pay attention to details, and consider the possible outcomes of their behaviors. These employees develop safe work habits and look for ways to improve.

Additionally, employees with positive attitudes tend to think before acting. These employees recognize potential hazards before they have time to cause problems, and then deal with them appropriately [39]. Another characteristic of a positive attitude is a person’s ability to focus on the task at hand, for example, always considering safety first when machinery and equipment are involved.

On the other hand, employees with a negative attitude complain about everything, including having to practice safety. They are less likely to care about the expected quality of the work or how they accomplish a given task. In other words, negative work attitudes can lead to unsafe working habits and accidents. Negative attitudes can force people into dangerous situations, causing them to lose interest or maintain pride in their poor work habits. Negative attitudes about safety will reduce employee safety, morale, quality, profitability, and business reputation, and increase production costs.

The discussion is compared with the previous study results conducted by Al-Rasheed et al. in 2021 [40], which explained that 98.6% had an overall positive attitude of safety and security workers toward good and safe COVID-19 prevention practices. This is in line with the results of this study which show that a good safety attitude or worker behavior at the workplace will provide good readiness and practice for preventing COVID-19. In contrast, other comparisons in research conducted by Zebley et al. in 2021 [41] explained that affective responses also connect with the emergence of stressors in COVID-19 healthcare workers. Indeed, it does not explicitly define the practice of preventing COVID-19 but describes the relationship between the two factors, namely, the affective domain and COVID-19 healthcare workers. This is corroborated by the results of this study which explain that preventing COVID-19 is in line with the affective domain of workers.

## 6. Conclusions

This study found that employees in the petrochemical industry have a positive degree of responses to COVID-19 prevention, safety attitudes, and the affective domain. The degree of implementation of COVID-19 prevention, safety attitude, and affective domain are not significantly dependent on employment characteristics such as gender, age, position, and work experience. The implementation of safety behavior appears to be evenly accepted and understood across employees from varying categories. Ultimately, a positive affective domain of employees is followed by a positive safety attitude; thus, effective COVID-19 prevention was established in the workplace based on the perspectives and attitudes of the employees.

### 6.1. Theoretical Implication

From a theoretical point of view, we can highlight the implications of the results of this study. One’s affective factors can be observed not only in education but also in work. Affective factors can also be interpreted as worker behaviors. The affective domain can reflect the behavior of workers in their daily work.

### 6.2. Practical Implication

From a practical point of view, the affective domain of a worker can be observed through their behavior and personality. Like this study, affective domain observations were carried out by adopting an effective questionnaire that refers to a person’s behavior in the workplace. This is proven by observing workers’ behaviors in line with understanding and applying COVID-19 prevention in work activities.

### 6.3. Study Limitation

This study is based on a survey with a random sample of employees from the petrochemical industry who are already familiar with the stringent work rules in the workplace. Therefore, the affective domain’s contribution to safety behavior compared to the workplace/other fields is different. In other words, it cannot be generalized to all occupations. This is because the petrochemical industry has a high work discipline.

### 6.4. Future Suggestions

This research was conducted on workers in industries with a high work discipline so that the three identified factors (affective, COVID-19 prevention, and safety) were aligned with each other because of the work culture that has been built. Therefore, in the future, similar research can be carried out, especially in the affective domain of workers in other industrial segments, so that it can be compared with the results of this study.

## Figures and Tables

**Table 1:** Sociodemographic characteristics of participants.

Characteristics		Frequency (%)
Total respondents		618 (80)
Gender	Male	438 (70.9)
	Female	180 (29.1)
Age groups	≤30 years old	98 (15.9)
	31–40 years old	166 (26.9)
	41–50 years old	194 (31.4)
	51–60 years old	126 (20.4)
	≥61 years old	34 (5.5)
Position	Worker	275 (44.5)
	Engineer	198 (32.0)
	Supervisor	145 (23.5)
Work experience	≤5 years	186 (30.1)
	6–10 years	183 (29.6)
	11–20 years	133 (21.5)
	21–30 years	74 (12.0)
	≥30 years	42 (6.8)

**Table 2:** COVID-19 prevention answers.

Questions	Response	Frequency (%)	Category Response	Frequency (%)
I wash my hands often.	4	309 (50.0)	Positive	618 (100)
	5	309 (50.0)		
I wear a mask in the office and in public.	4	297 (48.1)	Positive	618 (100)
	5	321 (51.9)		
I care about physical distancing by 2 m from others.	4	306 (49.5)	Positive	618 (100)
	5	312 (50.5)		
I avoid non-essential trips to the community.	4	298 (48.2)	Positive	618 (100)
	5	320 (51.8)		

**Table 3:** Safety attitude descriptive.

Question	Response	Frequency (%)	Category Response	Frequency (%)
I recognize the importance of safety in my workplace.	3	214 (34.6)	Neutral	214 (34.6)
	4	200 (32.4)	Positive	404 (65.4)
	5	204 (33.0)		
I encouraged my colleagues to participate in safety activities.	3	220 (35.6)	Neutral	220 (35.6)
	4	186 (30.1)	Positive	398 (64.4)
	5	212 (34.3)		
I am studying new knowledge regarding safety continuously.	3	215 (34.8)	Neutral	215 (34.8)
	4	207 (33.5)	Positive	403 (65.2)
	5	196 (31.7)		
I am trying to solve the conflicts among colleagues.	3	223 (36.1)	Neutral	223 (36.1)
	4	189 (30.6)	Positive	395 (63.9)
	5	206 (33.3)		
I am frequently communicating safety issues to colleagues.	3	215 (34.8)	Neutral	215 (34.8)
	4	195 (31.6)	Positive	403 (65.2)
	5	208 (33.7)		
I regularly provide colleagues with safety information.	3	205 (33.2)	Neutral	205 (33.2)
	4	205 (33.2)	Positive	413 (66.8)
	5	208 (33.7)		
I am willing to maintain the function of safety facilities.	3	185 (29.9)	Neutral	185 (29.9)
	4	227 (36.7)	Positive	433 (70.1)
	5	206 (33.3)		
While working it is very unlikely for me to get in contact with hazardous materials.	1	326 (52.8)	Positive	618 (100)
	2	292 (47.2)		
I know the proper procedures if a fire breaks out.	3	210 (34.0)	Neutral	210 (34.0)
	4	174 (28.2)	Positive	408 (66.0)
	5	234 (37.9)		

**Table 4:** Employee affective domain.

Grouping Affective Domain	N	Mean Response	Categories Response
Employee valuation of interdisciplinary and SE processes	618	3.63	Positive
Employee valuation of teamwork	618	3.48	Positive
Employee valuation of understanding of systems structure, hierarchy, and boundaries	618	3.53	Positive
Employee valuation of understanding relationships	618	3.88	Positive
Employee valuation of managerial skills andbig picture thinking	618	3.01	Neutral

**Table 5:** Mann–Whitney test (non-parametric differential test of two groups).

Characteristics	Frequency (%)	COVID-19 Prevention Score (Mean Rank)	Sig. 2-Tailed	Safety Attitude Score (Mean Rank)	*p*-Value	Affective Domain Score (Mean Rank)	Sig. 2-Tailed
Gender							
Male	438	310.63	0.799	304.98	0.323	310.54	0.821
Female	180	306.75		320.49		306.97	

Sig. < 0.05.

**Table 6:** Kruskal–Wallis test (non-parametric differential test of more than two groups).

Characteristics	Frequency (%)	COVID-19 Prevention Score (Mean Rank)	*p*-Value	Safety Attitude Score (Mean Rank)	*p*-Value	Affective Domain Score (Mean Rank)	*p*-Value
Age							
≤30 years	98	318.30	0.971	329.22	0.107	319.52	0.972
31–40 years	166	307.06		309.95		311.82	
41–50 years	194	306.04		315.63		304.53	
51–60 years	126	308.10		304.31		307.17	
≥61 years	34	321.00		234.71		306.31	
Position							
Worker	275	311.69	0.235	315.59	0.270	309.41	0.971
Engineer	198	321.12		292.95		307.59	
Supervisor	145	289.49		320.55		312.29	
Job experience							
≤5 years	186	315.83	0.684	315.95	0.576	320.62	0.378
6–10 years	183	299.27		316.20		300.42	
11–20 years	133	317.05		302.41		293.11	
21–30 years	74	319.50		313.55		337.53	
≥30 years	42	284.50		270.21		302.33	

Sig. < 0.05.

## Data Availability

The raw data supporting the conclusions of this article will be made available by the authors upon request, without undue reservation.
